# Subsurface Deformation Mechanism in Nano-cutting of Gallium Arsenide Using Molecular Dynamics Simulation

**DOI:** 10.1186/s11671-021-03574-3

**Published:** 2021-07-19

**Authors:** Chenghao Chen, Min Lai, Fengzhou Fang

**Affiliations:** State Key Laboratory of Precision Measuring Technology and Instruments, Laboratory of Micro/Nano Manufacturing Technology, Tianjin University, Tianjin, 300072 China

**Keywords:** Molecular dynamics, Gallium arsenide, Nano-cutting, Dislocation, Stacking fault, Phase transformation

## Abstract

During the nano-cutting process, monocrystalline gallium arsenide is faced with various surface/subsurface deformations and damages that significantly influence the product’s performance. In this paper, molecular dynamics simulations of nano-cutting on gallium arsenide are conducted to investigate the surface and subsurface deformation mechanism. Dislocations are found in the machined subsurface. Phase transformation and amorphization are studied by means of coordination numbers. Results reveal the existence of an intermediate phase with a coordination number of five during the cutting process. Models with different cutting speeds are established to investigate the effects on the dislocation. The effect of crystal anisotropy on the dislocation type and density is studied via models with different cutting orientations. In addition, the subsurface stress is also analyzed.

## Introduction

Gallium arsenide (GaAs) is a typical kind of III–V compound, and it is also one of the most important semiconductor materials. Favored by its great properties such as direct bandgap, high electron mobility and high resistivity, monocrystalline gallium arsenide finds wide applications in various areas such as infrared optical devices and microwave devices. With the continuous development of its production, the strict requirement of surface roughness, surface form accuracy, and subsurface damage are increasingly demanded. Nano-cutting technology is beneficial in improving the performance of gallium arsenide optical elements and in expanding the field of its applications. The nano-cutting process of gallium arsenide crystal is faced with many difficulties such as brittle crack, anisotropy, and other subsurface damages. Therefore, the study on the nano-cutting mechanism of gallium arsenide is of great significance in solving the machining problems, improving the surface quality of gallium arsenide, and developing related nanometric processing technology.

Many studies performed nanoscale experiments on gallium arsenide. Fang et al. [1] compared the nanoindentation and nanoscratch characteristics of gallium arsenide and silicon. Taylor et al. [2] investigated the ultra-low load nanoindentations in gallium arsenide (100) with a cube corner tip. Bradby et al. [3] found the pop-in events of gallium arsenide in a spherical indentation. Fang et al. [4, 5] investigated the machining properties of soft, brittle semiconductors and obtained gallium arsenide mirror surface for the first time by diamond turning. Although many studies about indentation and physical properties of gallium arsenide have been reported, little is known about its nano-cutting process and the mechanism of damage formation. This is mainly because of the great difficulty in conducting the experiments and subsequent characterizations. On the one hand, it is almost impossible to inspect the nano-cutting process and measure the nanometric data using on-line measurement due to the nanometric scale and the high cutting speed. On the other hand, it is costly to conduct a series of nanometric machining and measurement experiments.

Molecular dynamics (MD) simulation is one of the most efficient methods to explore the mechanism of nanometric machining. Shimada et al. [6, 7] proved that MD simulation is an effective way to describe the nanometric machining process. Komanduri et al. [8] found the structural transition phenomenon of silicon in the nano-cutting process with MD simulation. Pei et al. [9] studied the dislocation formation of copper with the large-scale MD models of nanometric machining. Lai et al. [10] studied the effects of critical rake angle and the material deformation of germanium in nanometric cutting and furthermore investigated the partially overlapped nano-cutting process [11]. In addition, multiscale modelling approach has developed a lot in the ultraprecision machining area [12–14]. The multiscale simulation combines simulation methods in different scales, such as MD model in nanoscale and FE model in nano/micro scale [15], to study the machining process in a more comprehensive and realistic way. In this paper, the subsurface deformation mechanism of nano-cutting process are focused, therefore the MD simulation is selected as the research method.

As for the studied materials, most MD simulation studies in nanometric machining focus on the element semiconductors such as silicon and germanium or metals such as copper and aluminum. Fewer studies have been reported about the numerical analysis on nano-cutting of gallium arsenide. Fan et al. [16] investigated the ductile response of gallium arsenide by MD simulation and turning experiments. Yi et al. [17] studied the phase transformation and anisotropic of gallium arsenide in nanoscratch process via MD simulation. In this paper, a series of three-dimensional MD simulations are carried out to investigate the ductile deformation in the process of nano-cutting on monocrystalline gallium arsenide. The effect of cutting parameters such as cutting speed and cutting orientation are also systematically studied.

## Methods

**Fig. 1:**
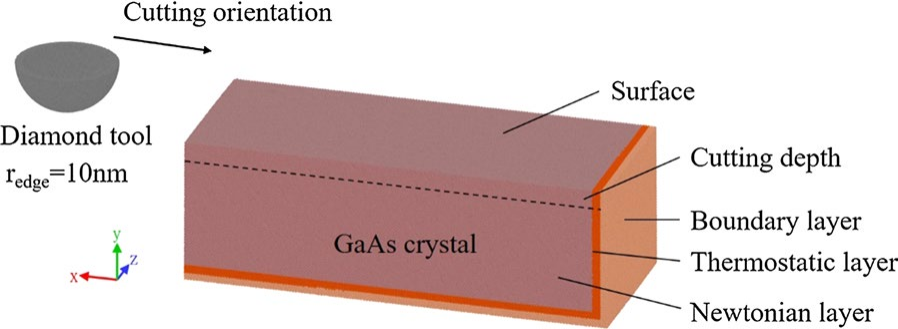
MD simulation model

MD simulation models are established to study the deformation behaviors of brittle crystal gallium arsenide in nano-cutting process. The three-dimensional MD simulation model is shown in Fig. 1. The workpiece is built as the monocrystalline gallium arsenide, crystallizing in the zincblende lattice with Ga and As atoms occupying the two FCC sublattices. The size of the workpiece is 85 nm × 30 nm × 35 nm. The workpiece model consists of three layers: boundary layer, thermostatic layer, and Newtonian layer. The workpiece is fixed by the boundary layer, while the thermostatic layer is set at a constant temperature of 293 K to imitate the heat dissipation in the real machining process. The Newtonian layer is the cutting area that will reveal the mechanism of the nano-cutting process. The motions of the atoms in the Newtonian layer obey the classical Newton’s second law. To imitate the diamond tool in the real cutting process, a hemisphere diamond tool model with a tool edge radius of 10 nm is built, and the cutting depth is set to 4 nm. The diamond tool is set at certain speeds to cut into the workpiece. With the limitation of computation resource, the model is minified and simplified, but it is still convincing to explain the ductile deformation and damage formation in nanometric scale. The diamond tool is simplified as a hemisphere to study the ductile removal behaviors on the horizontal direction like side-flow and pile-up on two sides of tool, which is the important characteristics of three-dimensional simulation. To study the effect of the process parameters, models with different cutting speeds and orientations are built. Table 1 lists the parameters of the models.

**Table 1:** Molecular dynamics simulation model parameters

Workpiece material	GaAs single crystal
Tool edge radius	10 nm
Workpiece size	85 nm × 30 nm × 35 nm
Lattice constant	5.65 Å
Initial temperature	293 K
Cutting depth	4 nm
Cutting speed	Cutting crystal orientation
200 m/s	$(010)\,[\overline{1}00]$
400 m/s	
800 m/s	
400 m/s	$(010)\,[\overline{1}00]$
	$$(\overline{1}10)\,[\overline{1}\overline{1}0]$$
	$$(\overline{2}11)\,[\overline{1}\overline{1}\overline{1}]$$

**Fig. 2:**
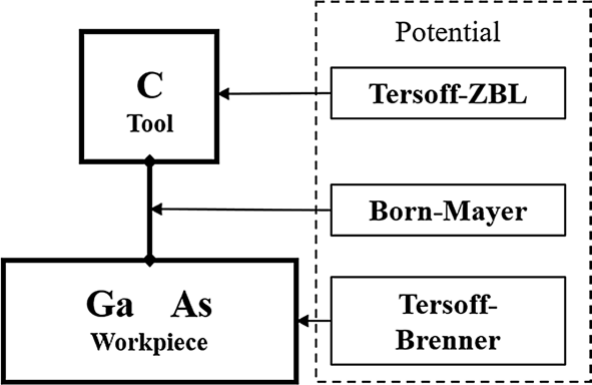
Potential system in the model

The potential is the basis for calculating the force and energy between atoms, and it is also one of the most important settings in MD simulation. In the previous study, a potential system consisting of three kinds of potential was determined for the simulation of gallium arsenide and diamond tool model [18]. In this system, a Tersoff-Brenner potential is used to describe the interaction between Ga and As, showing a good simulation effect [19]. An exponential repulsive potential is established to describe the interaction between the diamond tool and gallium arsenide workpiece via a quantum chemical method, which is in the form of a simplified Born–Mayer potential [18]. A Tersoff-ZBL potential is used in the diamond tool. The potential system is summarized in Fig. 2.

LAMMPS is used to carry out the MD simulation, while the visualization and analysis are via OVITO, including the dislocation analysis (DXA), stress calculation and coordination analysis. The relaxation of the workpiece is set as an NPT ensemble to minimize the system’s energy and stabilize the free surface, while the nano-cutting process is set as an NVE ensemble. The timestep for the integration in the simulation is 1.0 fs.

## Results and Discussion

### General Description

**Fig. 3:**
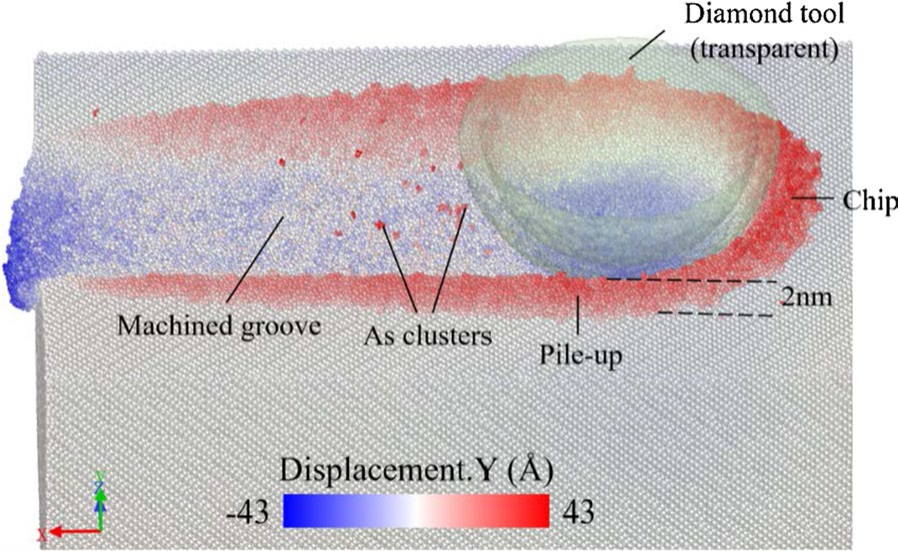
Nano-cutting simulation result colored with the atom’s displacement in the *y*-direction

As is shown in Fig. 3, the gallium arsenide workpiece is machined at a cutting distance of 60 nm with a cutting speed of 400 m/s along the [$\overline{1}$00] direction on the (010) plane. The workpiece is colored with the atom’s displacement in the *y*-direction. The atoms in front of the tool are observed to flow up, becoming chips by extrusion. A mount of atoms flow downwards and form the machined surface. On both sides of the machined groove, the atoms pile up with a height of 2 nm due to extrusion and plowing of the tool. The result revels that the deformation and removal of the brittle gallium arsenide crystal represents a ductile behavior when the machining is in nanometric scale. Few arsenide clusters precipitating to the machined surface are evident in the simulation process. This study reveals the presence of arsenide precipitation after annealing [20]. The Tersoff-Brenner potential was able to simulate the precipitation of arsenide clusters [19]. In the cutting process, the surface material is heated up with a cutting heat and then annealed, forming arsenide clusters. However, this precipitation is not a major concern in the nano-cutting process. Therefore, these atoms will be hidden in the subsequent analysis.

**Fig. 4:**
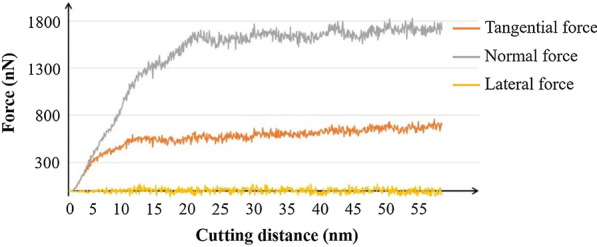
Cutting force in the nano-cutting process

Compare the cutting force curve during the machining process in Fig. 4. Initially, the tangential force and normal force rise with the tool cutting into the material. The lateral force fluctuates around 0 because the forces cancel out in the *z*-direction. When the forces are stabilized, the normal force and tangential force fluctuate around 1700 nN and 700 nN, respectively. It is found that the force in the *y*-direction is dominant in the cutting process because of the large effective negative rake angle of the diamond tool as presented in the model. The negative rake angle brings a large compressive stress, which causes a high normal force in the cutting process.

### Dislocations and Stacking Faults

The subsurface damage formation is an important part of the study of the nano-cutting mechanism. It is necessary to figure out the damage formation mechanism of gallium arsenide during machining to further optimize the processing parameters. Crystals have anisotropy on atomic structure, and it is one of the most serious problems in the machining process, which would affect the process performance with different orientations.

**Fig. 5:**
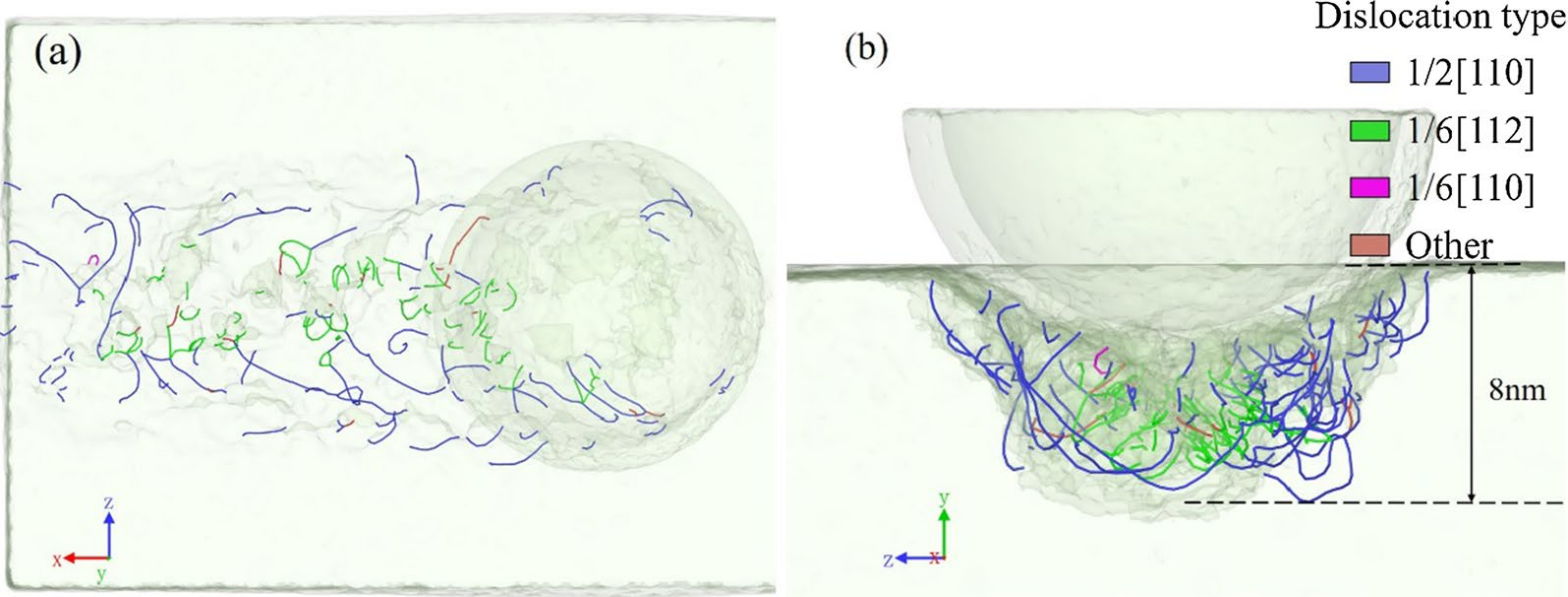
Perspective views of dislocation distribution in the machined subsurface, in **a** *y*-direction and **b** *x*-direction

The distribution of dislocations in the machined subsurface is shown in Fig. 5, and the tool motion is along (010) [$\overline{1}$00] cutting orientation. The dislocations are observed around the machined groove forming a dislocation layer of about 8 nm. Two main types of dislocations are present in the machined subsurface. The 1/2[110] dislocations mainly glide in the two sidewalls of the machined groove, while the 1/6[112] dislocations distribute under the groove bottom. The 1/2[110] and 1/6[112] dislocations proved to be perfect dislocations (blue lines) and partial dislocations (green lines), respectively. The formation of dislocations means the transitive motions of the local atoms, which implies that the deformation and removal are in the ductile state. It is proved that the brittle material also shows a ductile state at a sufficiently small scale.

**Fig. 6:**
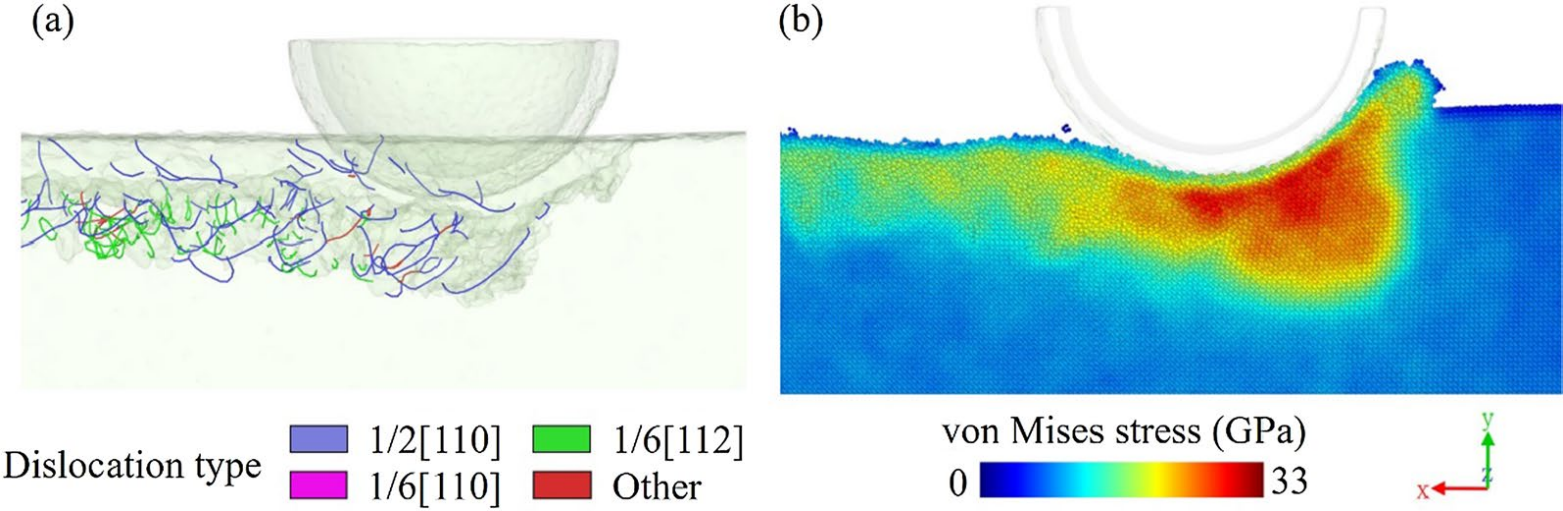
**a** Dislocation distribution and **b** von Mises stress distribution in the subsurface of the nano-cutting model

The von Mises stress, which is calculated including the shear stress components, is usually used to determine the formation of dislocation. Figure 6 is the cross-section view of the dislocation and the von Mises stress distribution. The high-stress area is concentrated under the tool because of extrusion. When the stress exerted by the tool exceeds the yield strength of the workpiece material, the material will slide along the slip surface, and the relative movement of the slip material will bring dislocations. Therefore, dislocations nucleate and extend into the single crystal because of high stress in the machining area. The local energy will be released by the atomic motion.

**Fig. 7:**
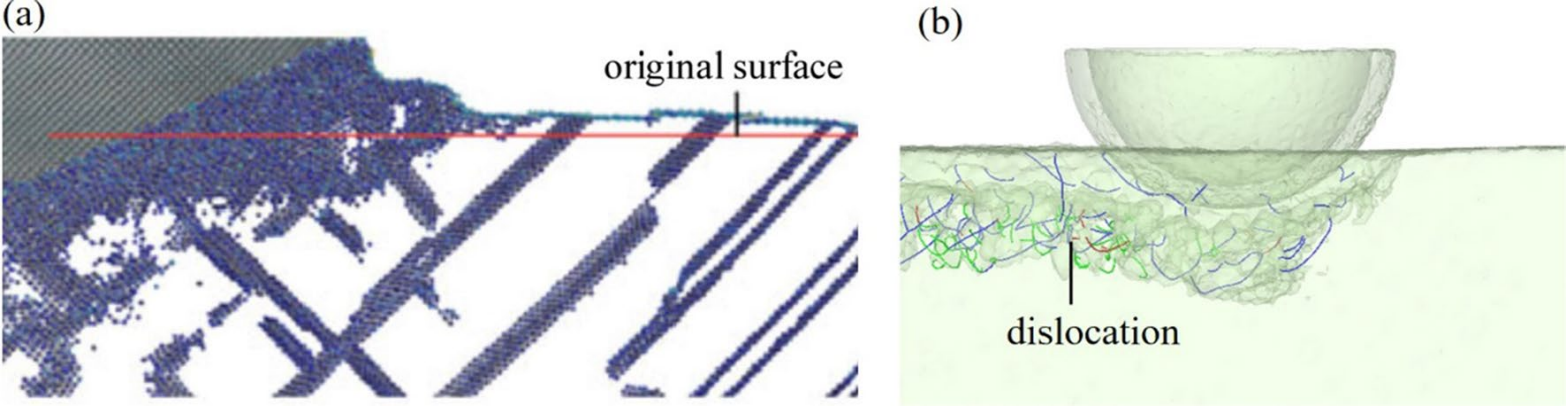
Dislocation in nano-cutting of different single-crystal materials: **a** copper [21] and **b** gallium arsenide

Dislocations in the brittle and ductile materials are greatly different. Gallium arsenide is a typical brittle crystal, and the dislocations are concentrated near the machined surface. However, dislocations in ductile materials like copper will extend and slip deep into the workpiece and form a high-density dislocation cluster during the cutting process [21], as shown in Fig. 7a. Dislocations result from the ductile deformation of materials. The extension of dislocation clusters for ductile materials leads to a wide range of ductile deformations and a diffused stress. However, the localization of dislocations in brittle materials such as gallium arsenide indicates that the ductile deformation only occurs near the surface without a complete release of stress. When the stress is concentrated to a threshold value, other kinds of damage, such as phase transformations or cracks, may appear. In our previous study [18], the crack formation of gallium arsenide was studied, and it’s found that the removal mode will change from ductile mode with dislocation formation to brittle mode with cracks when the cutting speed increases. The dislocation formation will release the stress which may cause crack initiation, and the deformation mode will tend to ductile instead of brittle in this instance.

**Fig. 8:**
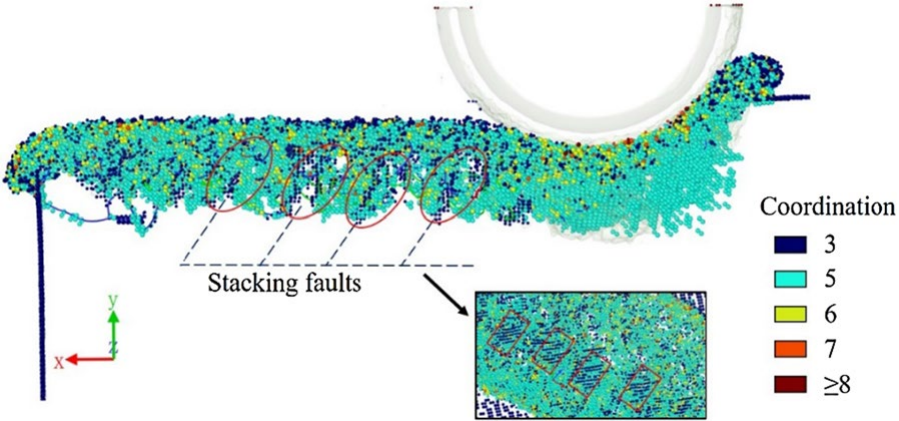
Stacking faults in the machined subsurface

Figure 8 reveals the stacking faults found in the machined subsurface. Atoms in the workpiece are colored based on the coordination number. For convenient inspection, four-coordination atoms with an original zincblende structure are hidden. It can be observed that there exist three-coordination atoms placed periodically in the machined subsurface. Combined with the partial dislocations found under the groove bottom, the partial dislocations that are mainly emitted from the grain boundaries or free surfaces may cause the formation of stacking faults or twinning [22, 23]. The stacking faults in the model are not completely dislocated atomic planes but several small areas in the boundary of single crystal and amorphous layer, therefore the partial dislocations exist at the boundaries of stacking faults. It can also be inferred that there may be stacking faults in the actual machined subsurface.

### Phase Transformation and Amorphization

The lattice structure of the material may change because of high stress during nano-cutting. By studying the phase transformation process, a better understanding of cutting gallium arsenide can be achieved, which is useful for optimizing the process parameters or in designing the pretreatment experiment before nano-cutting.

Monocrystalline gallium arsenide has a zincblende structure under normal pressure and temperature. However, the structure changes into a six-coordinate GaAs-II when the compressive stress reaches 17 GPa [24]. The phase transformation ends up with a wurtzite structure as stress increases beyond 60 GPa [25].

**Fig. 9:**
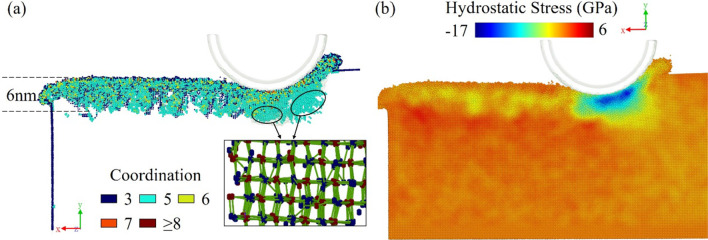
Distribution of **a** high coordination atoms and five-coordination structure and **b** hydrostatic stress distribution

Figure 9a presents the structural change in the model with a cutting speed of 400 m/s at the (010)[$\overline{1}$00] orientation. A layer of the machined area is observed to exhibit an amorphous structure with a thickness of about 6 nm without the characteristic structure of the crystal.

It is obvious that some atoms under the machining position turn to a five-coordination structure. Similar to the bct-5 structure of silicon crystal forming with compressive stress, this five-coordination structure is considered as an intermediate in the formation of the six-coordinate GaAs-I. Hydrostatic stress increases and causes the distortion of lattice with the tool cutting. However, as is shown in hydrostatic stress distribution of Fig. 9b, the maximum hydrostatic stress is 17 GPa, which just reaches the critical value of transformation from GaAs-I to GaAs-II. The subsurface area where the hydrostatic stress is higher than 17 GPa is very small, and most of the workpiece’s area has a hydrostatic stress below the transition threshold. As a result, it is hard to find large pieces of atoms with a six-coordination structure and the five-coordination structure is an intermediate phase from GaAs-I to GaAs-II.

**Fig. 10:**
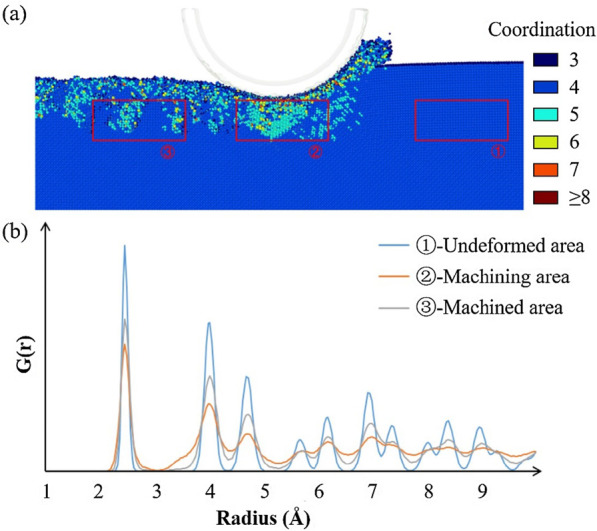
Selected area radial distribution function: **a** selected areas; **b** corresponding radial distribution function curves

Figure 10 depicts the radial distribution function curves of a selected area in the cutting process, which is the area beneath the diamond tool. The radial distribution curves before, during, and after processing are calculated. The workpiece has an ordered zincblende structure at the beginning, whose curve consists of regular peaks. When the diamond tool reaches over the selected area, the radial distribution function shows a disordered state both in the short range and long range. This implies the presence of a strong amorphization with the disordered arrangement of atoms in the area. However, the curve of the machined subsurface implies that the structure can recover and becomes ordered on the short range and disordered on the long range. With the release of stress, the material will change the structure to one with a minimum energy state. Atoms with an intermediate phase may change to amorphous or other state. Thus, the curve shows a stable machined state with amorphous gallium arsenide. It can be found that there is a side peak at 3.3 Å in the machining process, and the peak disappears when the tool leaves. This phenomenon reveals the formation and vanishment of the intermediate phase, which can be regarded as the elastic deformation in the cutting process. Without the force of tool, part of the atoms will get recovery to the zincblende structure, and the others will change to other stable phase or amorphous state, which represents the permanent ductile deformation.

### Cutting Speed and Orientation Effects on the Subsurface Deformation

The surface/subsurface damage formation is influenced by many factors, and different cutting parameters will affect the formation of the dislocation.

**Fig. 11:**
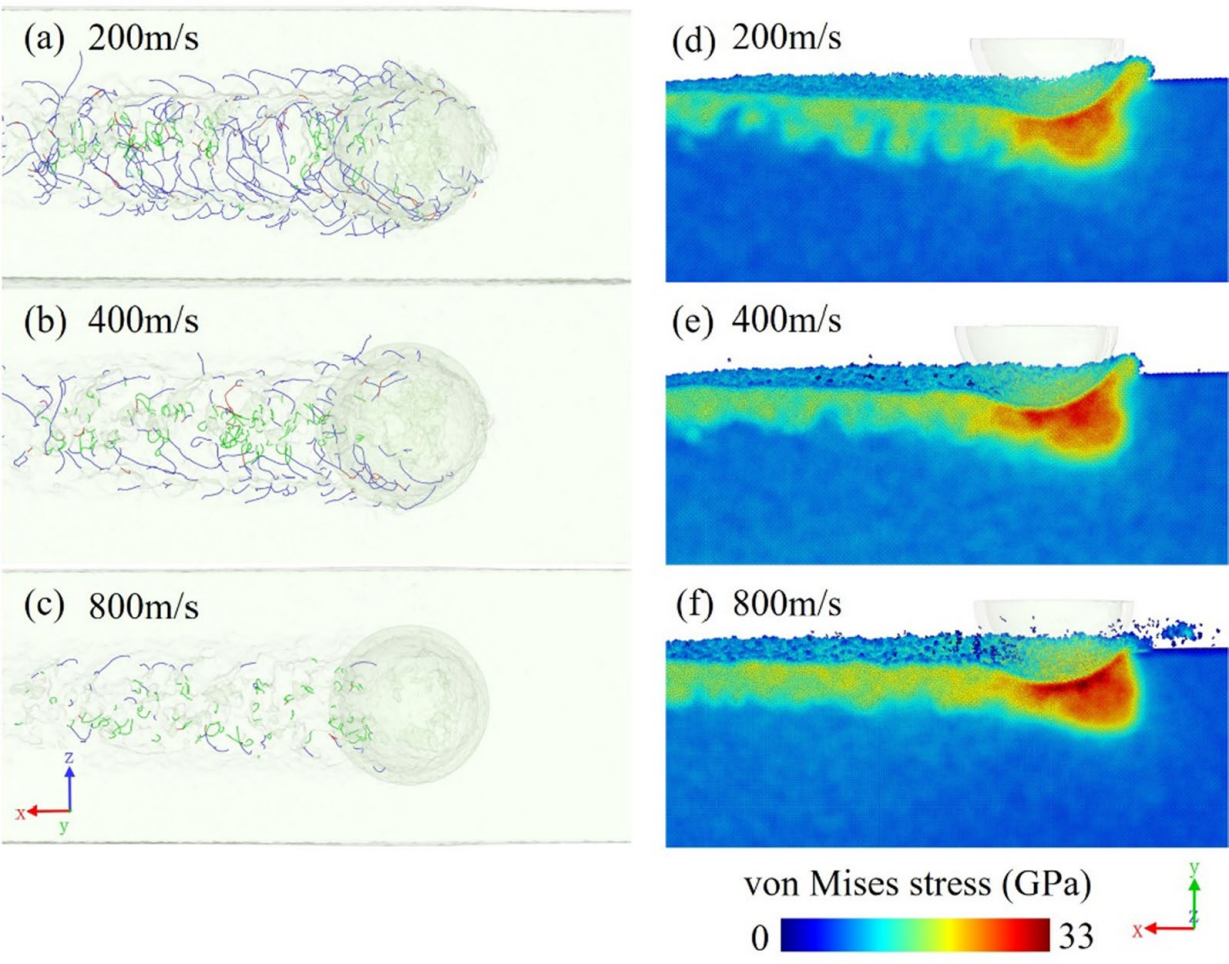
Perspective views of dislocation distribution and von Mises stress distributions in models with different cutting speeds of **a**, **d** 200 m/s, **b**, **e** 400 m/s, and **c**, **f** 800 m/s

The dislocation distributions with different cutting speeds are compared in Fig. 11a–c. The dislocation density gradually decreases as the cutting speed increases. The machined subsurface is richly supplied with dislocations, especially when the cutting speed is down to 200 m/s. When the cutting process is under a high strain rate, there is insufficient time for dislocation nucleation and propagation. As a result, there are less dislocations, and the ductile deformation which is induced by dislocations is also less dominant. The material will be deformed and disorganized rapidly, and the recovery time is also short, and amorphous atoms with non-regular structure will be easier to form. Therefore, a higher cutting speed is a way to restrain the formation of dislocations. Figure 11d–f are the cross-section views of the von Mises stress distribution in the three corresponding models. At a higher cutting speed, the stress of the material near the tool is relatively higher because of the large cutting energy. Comparing the three models, the 800 m/s model has a higher stress concentration in front of the tool, and the machined subsurface presents a more continuous stress region with higher values. On the contrary, the stress is released because of the formation of dislocations in the low-speed models. The ductile deformation that is induced by the dislocations will release the local strain energy and the stress that is caused by the bending and elastic deformation of the lattice. Therefore, in the workpiece with a higher cutting speed, the stress state can be severe because of the lack of dislocations, making it easier to form cracks in the cutting process.

**Fig. 12:**
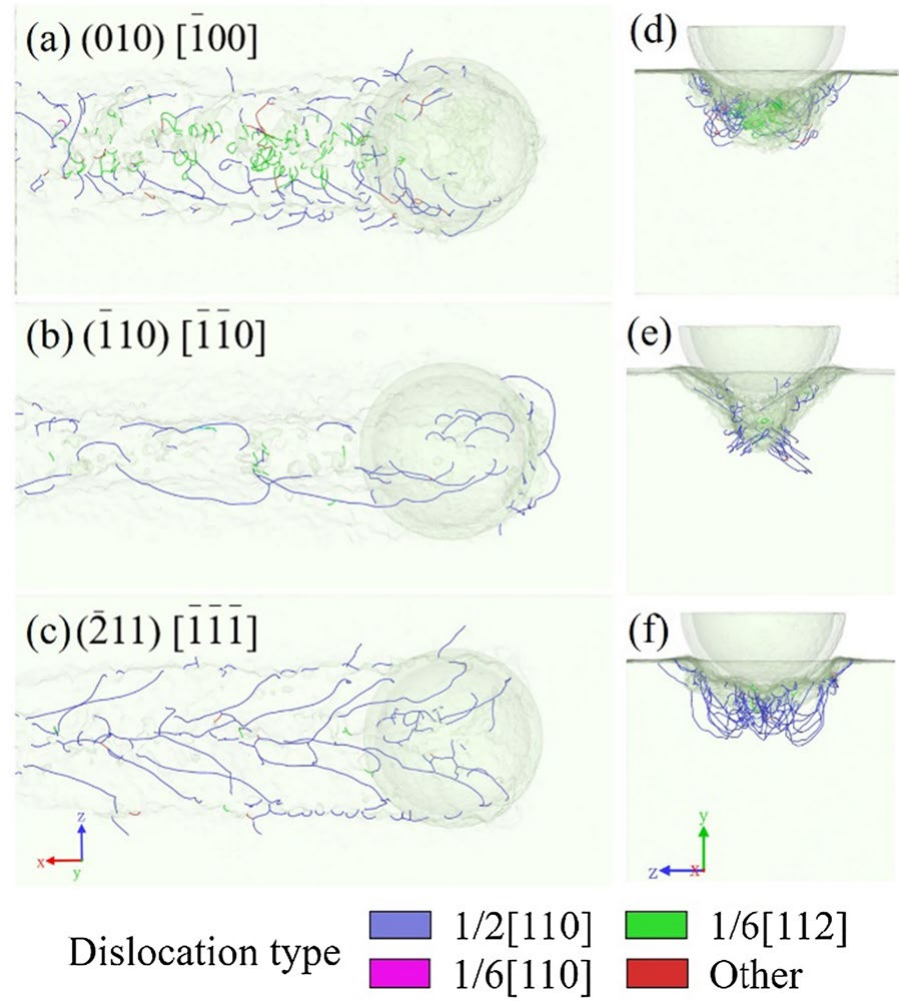
Perspective views of the dislocation distribution in the models with cutting orientations along **a**, **d** $(010)\,[\overline{1}00]$, **b**, **e** $(\overline{1}10)\,[\overline{1}\overline{1}0]$, and **c**, **f** $(\overline{2}11)\,[\overline{1}\overline{1}\overline{1}]$

Anisotropy is a serious problem in the nano-cutting process of the crystal [26, 27], including the single-crystal gallium arsenide. Due to its single-crystal structure, gallium arsenide crystal shows distinct properties in different crystal orientations. Three models with different cutting planes and orientations are built to study the anisotropy. The cutting orientations are (010)[$$\overline{1}$$00], ($$\overline{1}$$10)[$$\overline{1}$$$$\overline{1}$$0], and $$(\overline{2}11)\,[\overline{1}\overline{1}\overline{1}]$$. Figure 12a–f reflect the great differences that exist among the dislocation distributions. As stated above, there exist two kinds of dislocations in the (010)[$$\overline{1}$$00] model. When the cutting direction is along the $$(\overline{1}10)\,[\overline{1}\overline{1}0]$$ direction, most of the dislocations are at the bottom of the machined groove, while some clustered dislocations extend to the surface in front of the tool. Few dislocations are found on both sides of the sidewall in Fig. 12b. At the $$(\overline{2}11)\,[\overline{1}\overline{1}\overline{1}]$$ cutting orientation, tree-like dislocations that extend from the middle to both sides are observed, covering the machined subsurface as revealed in Fig. 12c. However, unlike the first model, there are few partial dislocations in the $$(\overline{1}10)\,[\overline{1}\overline{1}0]$$ and $$(\overline{2}11)\,[\overline{1}\overline{1}\overline{1}]$$ models, and most of the dislocations are perfect dislocations along the [110] orientation. Similarly, stacking faults are not found in the machined area of $$(\overline{1}10)\,[\overline{1}\overline{1}0]$$ and $$(\overline{2}11)\,[\overline{1}\overline{1}\overline{1}]$$ models, corresponding to the lack of partial dislocations. This also proves the effect of anisotropy in the material.

**Fig. 13:**
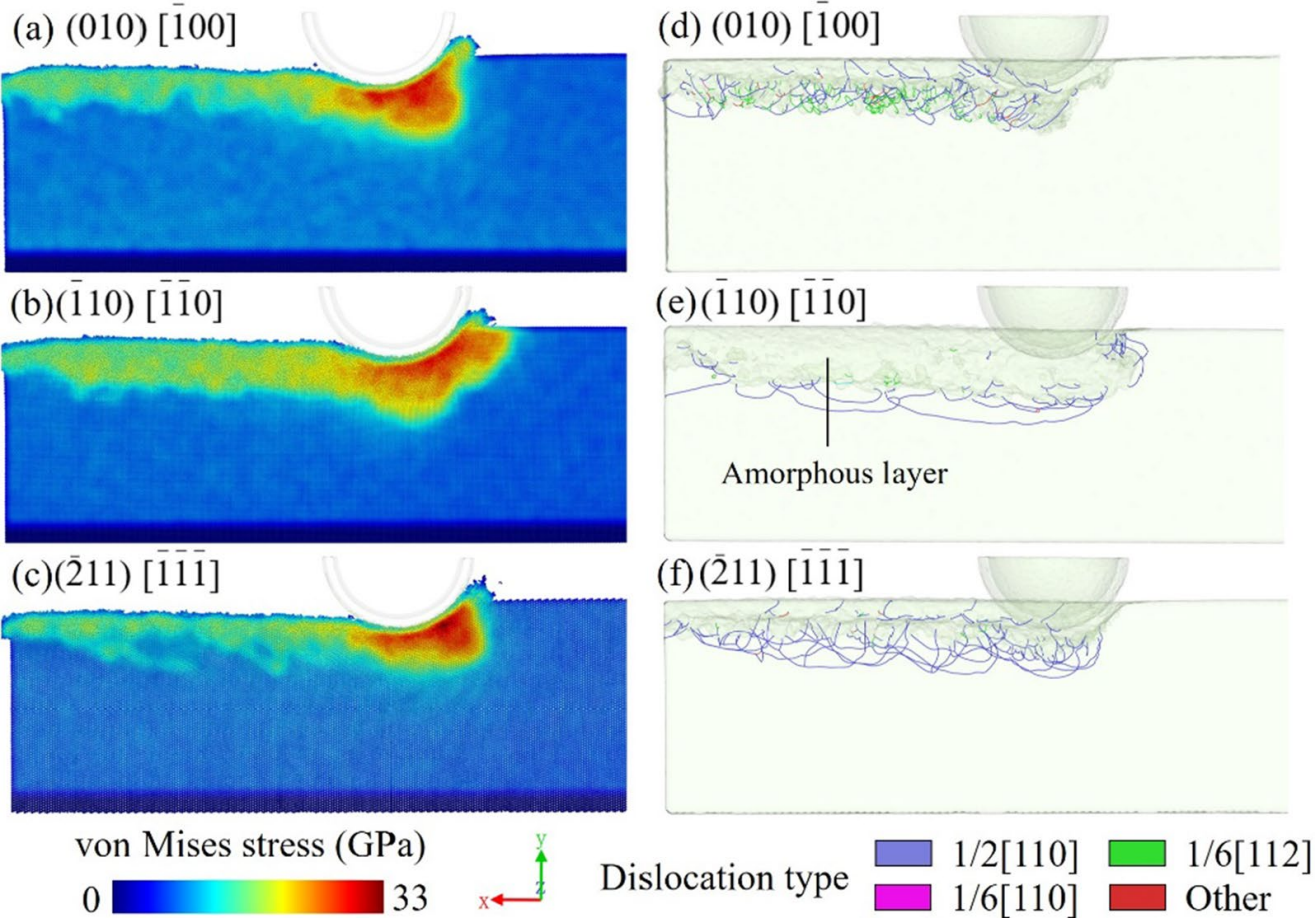
Von Mises stress distribution and dislocation distribution in the models with cutting orientations along **a**, **d** $(010)\,[\overline{1}00]$, **b**, **e** $(\overline{1}10)\,[\overline{1}\overline{1}0]$, and **c**, **f** $(\overline{2}11)\,[\overline{1}\overline{1}\overline{1}]$

Figure 13a–f compare the dislocation distribution and von Mises stress distribution of the three models with different cutting orientations. The crystal orientation has an obvious effect on stress propagation. In the (010)[$\overline{1}$00] and $(\overline{2}11)\,[\overline{1}\overline{1}\overline{1}]$ models, the stress is concentrated in front of the diamond tool, but the extending directions are different. However, the concentrated stress area of the $(\overline{1}10)\,[\overline{1}\overline{1}0]$ model is wider, and the region of high stress in front of the tool extends to the surface. Because of the difference in crystal orientations, the stress components along the slip direction will be different, resulting in the different appearances of dislocations. When the angle of the stress direction and slip surface is small, the slip is more likely to appear and the material will deform in ductile mode. On the contrary, the crack and brittle fracture tends to initiation when the ratio of the tensile stress perpendicular to the cleavage plane to the shear stress along the slip plane increases.

Comparing the stress and dislocation distributions, the models with a higher dislocation density may have lower stress in the machined subsurface. Figure 13e shows a thicker amorphous damage layer in the subsurface of the $(\overline{1}10)\,[\overline{1}\overline{1}0]$ model, which corresponds to the dense dislocation regions in the other models. This phenomenon shows that anisotropy may have an effect on the damage types. In the $(\overline{1}10)\,[\overline{1}\overline{1}0]$ model, the concentrated stress is hard to be released because of the less dislocation formation. As a result, the arrangement of atoms is disturbed under a severe stress state. The stress range is larger in the machined subsurface and the residual stress is higher. On the contrary, the $(\overline{2}11)\,[\overline{1}\overline{1}\overline{1}]$ model has a thinner stress layer in the machined subsurface due to the larger dislocation density. The formation of dislocation releases part of the stress during the cutting process. In the previous study [18], when the ductile damage mode turns from dislocation to amorphization, the machined subsurface exhibits a severe stress state and amorphous damage. The subsurface cracks are easier to form in the boundary of the amorphous and single crystal. It can be inferred that the cracks are more likely to occur when the cutting orientation is along the $(\overline{1}10)\,[\overline{1}\overline{1}0]$ direction. The formation of dislocation may reduce the brittle damage formation. In addition to the cleavage mechanism, it is also a reason why crack formation is affected by anisotropy.

In the cutting process, the material near the cutting tool receives high stress and severe compression, which will cause the material structural changes such as phase transformation and amorphization. Under the amorphous layer, slip will occur in a larger area and dislocations will form in the single crystal due to the widespread stress effects. These are the main ductile deformation in the nano-cutting of gallium arsenide. Different cutting condition will affect the local stress state and the ease of dislocation formation. When the dislocations is difficult to generate due to the high processing speed or anisotropy, the mode of ductile deformation will tends to be amorphous dominated because of the amorphization caused by unreleased stress. The crack will also easy to form in this situation. On the contrary, dislocations will be the main component of ductile deformation if the conditions are suitable for the material slip.

## Conclusion

MD simulation is used to study the mechanism of damage formation in the nano-cutting process of gallium arsenide crystal. The atomic motion and cutting force of the process are also analyzed. The dislocation, stacking fault, and phase transformation are mainly studied as the surface/subsurface damages. The conclusions can be summarized as follows:

1. In the nano-cutting of gallium arsenide, the dislocation and structural transformation are found as the main deformation mechanism in the machined subsurface.
2. Dislocations and stacking faults are observed in the machined subsurface groove, and the formations of stacking faults and partial dislocations are consistent.
3. An intermediate phase with five-coordination is found in front of the tool because hydrostatic stress is close to but not higher than the transition threshold (17 GPa). An amorphous layer forms in the machined subsurface.
4. With increasing cutting speed, the dislocation density decreases because of the high strain rate. Anisotropy has a great effect on the dislocation type and density. Partial dislocations are easier to form in the (010) [$\overline{1}$00] model. Moreover, the $(\overline{1}10)\,[\overline{1}\overline{1}0]$ model has a lower dislocation density and a more severe amorphization.

## Data Availability

The datasets generated or analysed during the current study are not publicly available due the data also forms part of an ongoing study, but are available from the corresponding author on reasonable request.

## Abbreviations

MD: Molecular dynamics
GaAs: Gallium arsenide
FE: Finite element
FCC: Face centered cubic
Tersoff-ZBL: Tersoff-Ziegler–Biersack–Littmark
LAMMPS: Large-scale atomic/molecular massively parallel simulator
OVITO: Open visualization tool
NPT: Number-pressure–temperature, isothermal-isobaric ensemble
NVE: Number-volume-energy, microcanonical ensemble
